# Molecular Characterization and Expression Patterns of *Sox3* and *Sox30* Genes and Response to Exogenous Hormones in the Chinese Soft-Shelled Turtle (*Pelodiscus sinensis*)

**DOI:** 10.3390/genes16111249

**Published:** 2025-10-22

**Authors:** Kailin Xiao, Yue Li, Tong Ren, Ziman Wang, Junxian Zhu, Chen Chen, Liqin Ji, Xiaoli Liu, Xiaoyou Hong, Chengqing Wei, Haigang Chen, Xinping Zhu, Xiaofang Lai, Wei Li

**Affiliations:** 1 College of Marine Science and Fisheries, Jiangsu Ocean University, Lianyungang 222005, China; xkl954334197@163.com; 2 Key Laboratory of Tropical & Subtropical Fishery Resource Application & Cultivation of Ministry of Agriculture and Rural Affairs, Pearl River Fisheries Research Institute, Chinese Academy of Fishery Sciences, Guangzhou 510380, China; liyue20001215@163.com (Y.L.); zhujunxian_1994@163.com (J.Z.); chenchen@prfri.ac.cn (C.C.); jiliqin@prfri.ac.cn (L.J.); liuxl@prfri.ac.cn (X.L.); hxy@prfri.ac.cn (X.H.); wcq1970@163.com (C.W.); harbourchen66@163.com (H.C.); zhuxinping@prfri.ac.cn (X.Z.); 3 Science and Technology Research Center of China Customs, Beijing 100026, China; blue2603@163.com; 4 School of Marine and Fisheries, Guangdong Eco-Engineering Polytechnic, Guangzhou 510520, China; wang_ziman@163.com

**Keywords:** *Pelodiscus sinensis*, *Sox3*, *Sox30*, exogenous hormones, gonadal differentiation and development

## Abstract

Background/Objectives: The Sox transcription factor family is critical for gonadal development and sex differentiation in animals, yet its roles in chelonians, particularly in the Chinese soft-shelled turtle (*Pelodiscus sinensis*), have rarely been investigated. Methods: This study cloned and analyzed the cDNA sequences of *Sox3* and *Sox30* genes from *P. sinensis*, examining their amino acid sequences and structural properties. Real-time quantitative PCR (RT-qPCR) was used to assess the expression of these two genes in different adult tissues and at various stages of embryonic gonadal development. Additionally, the effects of exogenous hormones (17β-estradiol, E_2_ and 17α-Methyltestosterone, MT) on the expression of *Sox3* and *Sox30* were also investigated. Results: The results indicated that *Sox3* showed significantly elevated expression in female gonads, kidney, brain, liver, lung, spleen, and muscle relative to male counterparts, displaying a female-biased expression pattern. In contrast, *Sox30* showed a male-biased pattern, with higher expression in male gonads, spleen, muscle, brain, and liver than in females, showing expression. Both genes were expressed at low levels. Exogenous hormone treatments revealed that MT significantly downregulated *Sox3* expression in female embryos, whereas E_2_ significantly enhanced *Sox3* expression in male embryos. Furthermore, MT treatment significantly upregulated *Sox30* expression in female embryos, and E_2_ treatment also significantly increased *Sox30* expression in male embryos. Conclusions: These findings suggest that *Sox3* and *Sox30* play crucial roles in the gonadal development of *P. sinensis*, with *Sox3* potentially involved in ovarian development and *Sox30* in testicular maturation. Both genes are regulated by exogenous hormones, highlighting their importance in sex differentiation and gonadal development. This study provides valuable theoretical insights for further exploration of the molecular mechanisms of sex regulation in reptiles.

## 1. Introduction

Members of the *Sox* gene family are transcription factors defined by a conserved high-mobility group (HMG) box that mediates DNA binding, and they are involved in processes such as early embryonic development, gonadal differentiation, and nervous system development [1]. As major members of this family, the *Sox3* and *Sox30* genes were first identified in humans (*Homo sapiens*) and mice (*Mus musculus*) [2]. Previous studies have shown that *Sox3* mRNA plays a role in neural crest development and adult gonadal differentiation in *M*. *musculus*. Furthermore, *Sox3* expression levels have been reported to be higher in the ovaries than in the testes of Japanese eel (*Anguilla japonica*) [3], Japanese flounder (*Paralichthys olivaceus*) [4], and Nile tilapia (*Oreochromis niloticus*) [5]. Knockout of the *Sox3* gene in *M*. *musculus* has been shown to impair germline stem cell differentiation during development [6]. Similarly, Hong et al. [7] demonstrated that *Sox3* knockout in zebrafish (*Danio rerio*) led to delayed follicular development and reduced fecundity in females. *Sox30* is the sole member of the Sox H subfamily. In *M*. *musculus*, *Sox30* expression exhibits remarkable tissue specificity, being abundantly expressed only in the adult male testis, predominantly in spermatocytes and spermatogonia. Its expression increases significantly at postnatal day 21, the period when spermatocytes first appear, suggesting that *Sox30* may be involved in spermatid differentiation during the post-meiotic haploid stage [8]. Wei et al. [9] reported that the *Sox30* gene is specifically expressed in the testes of *O*. *niloticus*. The *Sox30* gene is primarily involved in male germ cell maturation, and studies have shown that its knockout its inhibits sperm formation while having no effect on oocyte formation or meiosis [10,11]. Fei Han et al. [12] reported that loss of the *Sox30* can cause non-obstructive azoospermia (NOA), whereas re-expression of *Sox30* rescues spermatogenesis and restores fertility, consistent with observations in *Sox30*-deficient mice. In the transcriptomic analysis of gonadal differentiation in the Chinese soft-shelled turtle (*Pelodiscus sinensis*), *Sox3* and *Sox30* were found to be highly expressed in the ovary and testis, respectively [13]. However, their potential functions in the gonadal differentiation and development of *P. sinensis* remain unclear.

*P. sinensis*, belonging to the class Reptilia, order Testudines, family Trionychidae, and genus *Pelodiscus*, is a high-value, is high-value “famous, special, and high-quality” aquaculture species in China. It is widely favored for its medicinal and edible value [14]. *P. sinensis* exhibits pronounced sexual dimorphism, with males growing faster than females under the same conditions. Consequently, there is demand for all-male breeding in the aquaculture industry. Identifying and studying genes related to gonadal differentiation and development may provide molecular targets for sex-controlled breeding in aquatic animals. In addition, previous studies have demonstrated that sex hormones play an important role in gonadal differentiation and development. 17β-Estradiol (E_2_) is a natural estrogen [15]. 17α-Methyltestosterone (MT) has been synthesized as an androgen agonist [16]. Both have been widely used in studies related to gonadal differentiation and development. Pieau et al. [17] reported that injecting of E_2_ into European pond turtles (*Emys orbicularis*) at 25 °C induces male-to-female sex reversal. El-Greisy et al. reported that treatment with MT induced masculinization of gonadal development in female juveniles of the red-spotted grouper (*Epinephelus akaara*) [18] and *O. niloticus* [19]. However, whether E_2_ and MT regulate the expression of *Sox3* and *Sox30* in *P*. *sinensis*, thereby participating in gonadal differentiation and development, remains unclear. Given the aforementioned background, this study cloned the cDNA sequences of *Sox3* and *Sox30* genes, analyzed their sequence characteristics, examined their expression patterns across different adult tissues and in gonads at various embryonic developmental stages, and evaluated their expression responses to exogenous hormone treatments (E_2_ and MT). These results will provide fundamental data for further elucidating the physiological roles of *Sox3* and *Sox30* in gonadal differentiation and development of *P. sinensis*.

## 2. Materials and Methods

### 2.1. Collection of Experimental Materials

*P. sinensis* used in this study were purchased from Caixing Industrial Co., Ltd. (Huizhou, China). Three healthy, three-year-old *P. sinensis* were selected, anesthetized, and euthanized by exsanguination. Heart, liver, brain, kidney, spleen, muscle, ovary, and testis tissues were collected from each individual, placed into RNase-free cryogenic tubes, rapidly frozen in liquid nitrogen, and stored at −80 °C until use. Three biological replicates were obtained for each tissue type. Additionally, 1000 fertilized *P. sinensis* eggs were collected, and gonadal tissues were sampled at developmental stages 14, 16, 18, 20, 22, 24, and 26. Embryonic staging was performed according to morphological characteristics described in a previous study [20], and other tissues from the embryos were collected for sex identification. At each stage, 120 embryos were collected, and based on the results of sex identification (Section 2.2), gonads from 20 male or female embryos were pooled into one tube, with three biological replicates per stage. Fertilized eggs were incubated using a medium-free method [21] under controlled conditions at 31 ± 1 °C and humidity of 75–85%.

### 2.2. DNA Extraction and Embryonic Sex Identification

DNA was extracted from *P*. *sinensis* embryonic samples following the instructions provided with the kit (ONREW, Shanghai, China). The extracted DNA was collected and stored at −20 °C until use. PCR amplification was performed using the *P. sinensis* species-specific primer pair PB1 [22] (Table 1). The 20 μL reaction system consisted of 7 μL of double-distilled water (ddH_2_O), 1 μL of PB1-F, 1 μL of PB1-R, 1 μL of genomic DNA template (50 ng/μL), and 10 μL of 2× Accurate Taq Master Mix. The amplification program was as follows: initial denaturation at 94 °C for 30 s; 35 cycles of denaturation at 98 °C for 10 s, annealing at 60 °C for 30 s, and extension at 72 °C for 60 s; and a final extension at 72 °C for 120 s. The resulting products were analyzed via 1% agarose gel electrophoresis to determine the genetic sex of the embryos.

### 2.3. Total RNA Extraction and First-Strand cDNA Synthesis

Total RNA was isolated from various tissues of *P. sinensis* using Trizol reagent (Takara, Beijing, China) according to the manufacturer’s protocol. RNA concentration and quality were assessed using the NanoQ™ nucleic acid analyzer (Thermo Scientific, Madison, WI, USA), and 1% agarose gel electrophoresis. Genomic DNA was removed using the HiScript^®^ III RT SuperMix for qPCR (+gDNA wiper) kit (Vazyme, Nanjing, China). First-strand cDNA was synthesized following the manufacturer’s instructions, and the resulting cDNA was stored at −20 °C.

### 2.4. Cloning of Sox3 and Sox30 Genes in P. sinensis

Based on the *P. sinensis Sox3* gene sequence (GenBank accession no. XM_006115453.3) and *Sox30* gene sequence (GenBank accession no. XM_006138296.4) available in the NCBI database, specific primer pairs (*Sox3*-F/*Sox3*-R and *Sox30*-F/*Sox30*-R; Table 1) were designed. The open reading frame (ORF) regions of *Sox3* and *Sox30* were amplified using cDNA from the ovaries and testes of three-year-old *P. sinensis* as templates. PCR products were analyzed on 1% agarose gels, and target bands were excised and purified using a Gel Extraction Kit (Omega Bio-tek, Norcross, GA, USA). Purified fragments were ligated into the pMD19-T vector (TaKaRa) and transformed into Escherichia coli DH5α competent cells (TaKaRa). Positive clones were verified by colony PCR and submitted to Guangzhou Tianyi Huiyuan Gene Technology Co., Ltd. (Guangzhou, China) for sequencing.

### 2.5. Bioinformatic Analysis of Sox3 and Sox30 Genes

The open reading frames (ORFs) were predicted using the ExPASy online tool (https://web.expasy.org/translate/ (accessed on 30 October 2024)) [23]. The molecular weight (kDa), isoelectric point (pI), instability index, total fat content, and grand average of hydropathicity (GRAVY) of the Sox3 and Sox30 proteins were calculated using ProtParam software [24]. The hydrophobicity, structural domains, and phosphorylation sites of the *Sox3* and *Sox30* proteins were predicted using ProtScale, SMART, and NetPhos-3.1 [25], respectively. The secondary structures of Sox3 and Sox30 proteins were analyzed using SOPMA [26], and their tertiary structures were constructed via homology modeling with SWISS-MODEL [27]. Amino acid sequence translation and homology comparison were perform using DNAMAN 8 software [28] (Table 2). Phylogenetic analysis of the Sox3 and Sox30 protein sequences was carried out in MEGA 11 [29] using the neighbor-joining method with 1000 bootstrap replicates.

### 2.6. Tissue Expression Analysis

Specific quantitative primers for *Sox3* (*Sox3*-qF/*Sox3*-qR) and *Sox30* (*Sox30*-qF/*Sox30*-qR) were designed using Primer 5 software, with *Ef1*α [30] selected as the reference gene (Table 1). Real-time quantitative PCR (RT-qPCR) was performed using cDNA from different *P. sinensis* tissues as templates on an Applied Biosystems StepOnePlus Real-Time PCR System (Applied Biosystems, Singapore). Each 20 μL reaction mixture contained 10 μL of 2× iTaq Universal SYBR Green Supermix (Bio-Rad, Hercules, CA, USA), 7 μL of double-distilled water (ddH_2_O), 1 μL of cDNA template, and 1 μL each of forward and reverse primers. The cycling conditions were as follows: initial denaturation at 95 °C for 5 min; 40 cycles of 95 °C for 10 s, 60 °C for 20 s, and 72 °C for 20 s. Melting curve analysis was subsequently performed at 95 °C for 15 s, 60 °C for 60 s, and 95 °C for 15 s.

### 2.7. Exogenous Hormone Treatment of P. sinensis Embryos

A total of 2600 embryos, confirmed to be fertilized at 12 h post-oviposition, were randomly divided into three groups: MT treatment group, E_2_ treatment group, and control group (treated with 95% ethanol). E_2_ and MT were each dissolved in 95% ethanol at a concentration of 20 μg/μL [31]. Prior to gonadal differentiation (embryonic stages 12–14) [32], 5 μL of the dissolved E_2_ solution or MT was applied to the eggshell, with the treatment repeated for two consecutive days. The control group received the same volume of solvent (95% ethanol). Gonads were then randomly collected at embryonic stages 14, 16, 18, 20, 22, 24, and 26. The methods for fertilized egg incubation, gonad collection, and sex identification are detailed in Section 2.1 and Section 2.2.

### 2.8. Data Analysis

The relative expression levels of the target gene were calculated using the 2^−ΔΔCt^ method. Each experiment was performed with at least two independent replicates to ensure the reliability of the results. Statistical analysis was conducted using SPSS 22.0 software, and one-way analysis of variance (ANOVA) was applied to analyze differential expression in the following three comparisons: between males and females in the same tissue, between male and female embryos at the same stage, and between the treatment group and control group at the same stage. The criteria for determining significant differences were as follows: *p* < 0.05 indicated a significant difference, and *p* < 0.01 indicated an extremely significant difference. All data were presented as mean ± standard error of the mean (SEM).

## 3. Results

### 3.1. Cloning of Sox3 and Sox30 Genes in P. sinensis and Analysis of Their Deduced Amino Acid Sequences

I cloned the coding regions (CDS) of *Sox3* and *Sox30* genes, and aligned their sequences with the corresponding reference sequences in the NCBI database, respectively. The results showed that the cloned CDS sequence of *Sox3* gene had 100% sequence identity with the database sequence, and the cloned CDS sequence of *Sox30* gene also had 100% sequence identity with the database sequence (Figures S1 and S2).

**Figure 1:**
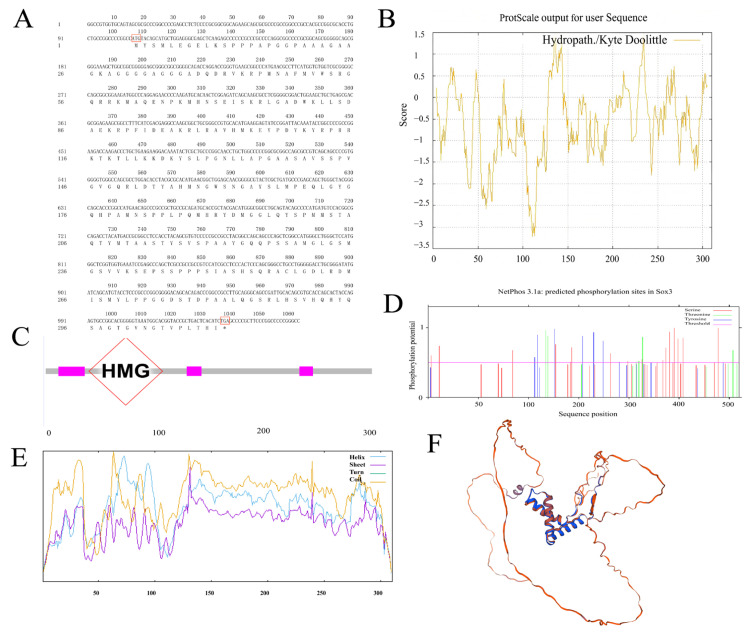
(**A**) Nucleotide sequence and deduced amino acid sequence of the *Sox3* gene in *P. sinensis*. (**B**) Hydrophilicity/hydrophobicity profile of *Sox3*. (**C**) *Sox3* protein domain. (**D**) Predicted phosphorylation sites of *Sox3*. (**E**) Predicted secondary structure of *Sox3*. (**F**) Predicted tertiary structure of *Sox3*. Notation: (**A**) ATG denotes the initiation codon, while TGA denotes the termination codon. (**C**) The gray bar represents the protein backbone; magenta boxes denote conserved motifs. The red diamond highlights the HMG box domain—the canonical DNA-binding domain of SOX family proteins—which recognizes specific DNA sequences and mediates transcriptional regulation. (**D**) Serine (Ser), Threonine (Thr), and Tyrosine (Tyr) indicate putative phosphorylation sites. (**E**) Blue (Helix): α-helix, Purple (Sheet): β-sheet, Green (Turn): β-turn, Orange (Coil): Random coil.

**Figure 2:**
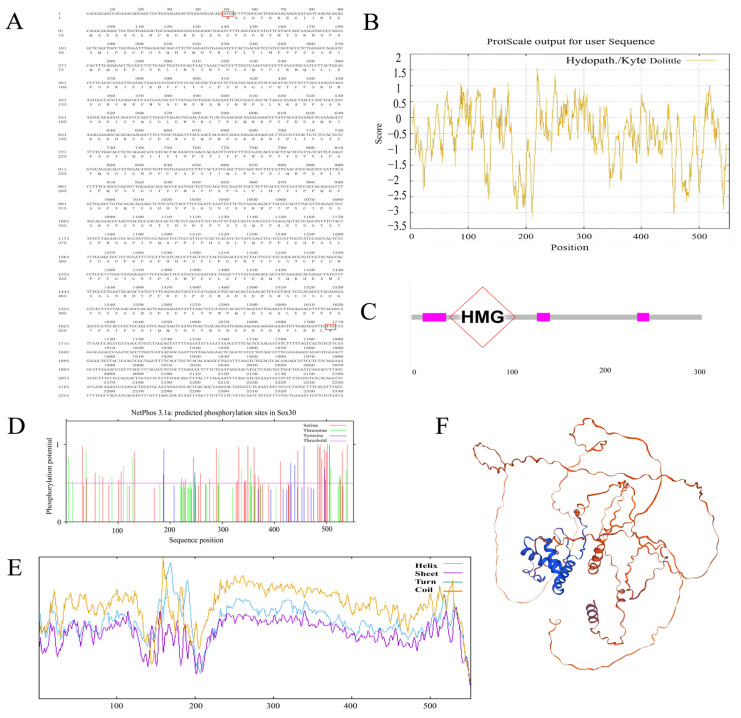
(**A**) Nucleotide sequence and deduced amino acid sequence of the *Sox30* gene in *P. sinensis*. (**B**) Hydrophilicity/hydrophobicity profile of *Sox30*. (**C**) *Sox30* protein domain. (**D**) Predicted phosphorylation sites of *Sox30*. (**E**) Predicted secondary structure of *Sox30*. (**F**) Predicted tertiary structure of *Sox30*. Notation: (**A**) ATG denotes the initiation codon, while TAA denotes the termination codon. (**C**) The gray bar represents the protein backbone; magenta boxes denote conserved motifs. The red diamond highlights the HMG box domain—the canonical DNA-binding domain of SOX family proteins—which recognizes specific DNA sequences and mediates transcriptional regulation. (**D**) Serine (Ser), Threonine (Thr), and Tyrosine (Tyr) indicate putative phosphorylation sites. (**E**) Blue (Helix): α-helix, Purple (Sheet): β-sheet, Green (Turn): β-turn, Orange (Coil): Random coil.

The cloned *Sox3* gene cDNA sequence is 1062 bp in length, with a coding sequence (CDS) of 933 bp that encodes 310 amino acids (Figure 1A). The molecular formula of the *P. sinensis Sox3* protein is C_1437_H_2266_N_424_O_437_S_20_. Its theoretical molecular weight is 33,115.58, theoretical isoelectric point (pI) is 9.66, and instability index (II) is 69.35, indicating that this protein is unstable. The aliphatic index of the *P. sinensis Sox3* protein is 57.10, with a grand average of hydrophilicity (GRAVY) of −0.597 (Figure 1B). The entire peptide chain of *P. sinensis Sox3* protein exhibits hydrophilic properties, so it is classified as a hydrophilic protein. The *P. sinensis Sox3* protein contains an HMG domain, which is a DNA-binding domain (Figure 1C). The *Sox3* protein of *P. sinensis* highly conserved and possesses 2 serine phosphorylation sites, 9 threonine phosphorylation sites, and 4 tyrosine phosphorylation sites (Figure 1D). Secondary structure analysis of the protein using the Protean program in DNAStar (Figure 1E) revealed that the secondary structure of the *P. sinensis Sox3* protein comprises 18.39% α-helix, 2.26% extended chain, and 79.35% disordered coil. The tertiary of the *Sox3* protein was predicted using SWISS-MODEL (Figure 2F). The results showed that the protein is mainly composed of α-helices, disordered coils, and extended chains, which is basically consistent with the secondary structure prediction.

Similarly, the full-length cDNA sequence of the *P. sinensis Sox30* gene was 2340 bp, with a CDS of 1659 bp that encodes 552 amino acids (Figure 2A). The molecular formula of the *P. sinensis Sox30* protein is C_2702_H_4149_N_741_O_811_S_16_, with a theoretical molecular weight of 60,503.11 Da, a pI of 6.52, and an instability index of 60.31, which also indicates that the protein is unstable. The aliphatic index of the *P. sinensis Sox30* protein was 63.61, and its grand average of hydrophilicity was −0.564 (Figure 2B), classifying it as a hydrophilic protein. Domain analysis identified a conserved HMG domain in this protein (Figure 2C). Phosphorylation site prediction of the *P. sinensis Sox30* protein indicated 6 serine, 17 threonine, and 6 tyrosine sites (Figure 2D). Secondary structure prediction revealed that the *P. sinensis Sox30* protein is composed of 11.78% α-helices, 1.09% extended chains, and 87.14% random coils (Figure 2E). The tertiary structure of the *P. sinensis Sox30* protein was predicted using SWISS-MODEL (Figure 2F). The results showed that the protein is mainly composed of α-helices, disordered coils, and extended chains, which is basically consistent with the secondary structure prediction.

### 3.2. Homology Alignment and Phylogenetic Tree Construction of the Sox3 and Sox30 Genes in P. sinensis

**Figure 3:**
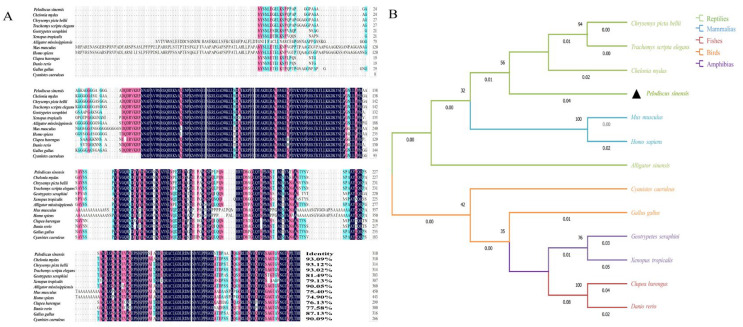
Multiple sequence alignment and phylogenetic tree analysis of *Sox3* amino acid sequences in *P. sinensis* and representative species. (**A**) Alignment of *Sox3* amino acid sequences. (**B**) phylogenetic tree of *Sox3* amino acid sequences. Notation: (**B**). The black triangle indicates *P. sinensis*, the target species of this study, which is marked as a key node in this phylogenetic tree.

Sequence homology analysis revealed that the *Sox3* protein of *P. sinensis* exhibited 93.09, 93.12, and 93.02% identity with green sea turtle (*C. mydas*), western painted turtle (*C. picta bellii*), and red-eared slider (*T. scripta elegans*), respectively. Sequence identities with chicken (*G. gallus*), Eurasian blue tit (*C. caeruleus*), western clawed frog (*X. tropicalis*), Gaboon caecilian (*G. seraphini*), and American alligator (*A. mississippiensis*) were 87.13, 90.09, 79.13, 81.49, and 90.05%, respectively. More distant similarities were observed with Atlantic herring (*C. harengus*, 76.13%), *D. rerio* (77.58%), *M. musculus* (75.40%), and *H. sapiens* (74.90%) (Figure 3A). The maximum likelihood (ML) phylogenetic tree indicated that *Sox3* from *P. sinensis* clustered most closely with other turtle species, followed by amphibians, mammals, and birds, while showing a more distant relationship with teleost fishes (Figure 3B).

**Figure 4:**
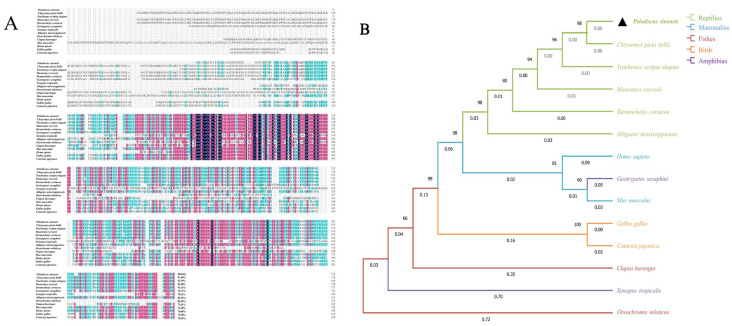
Multiple sequence alignment and phylogenetic tree analysis of *Sox30* amino acid sequences in *P. sinensis* and representative species. (**A**) Alignment of *Sox30* amino acid sequences. (**B**) phylogenetic tree of *Sox30* amino acid sequences. Notation: (**B**). The black triangle indicates *P. sinensis*, the target species of this study, which is marked as a key node in this phylogenetic tree.

Homology alignment revealed that the *Sox30* sequence of *P. sinensis* exhibited 91.40, 91.39, 94.40, and 91.33% homology with the western *C. picta bellii*, *T. scripta elegans*, Chinese pond turtle (*M. reevesii*), and leatherback sea turtle (*D. coriacea*), respectively. Sequence identities with the *A. mississippiensis*, Japanese quail (*C. japonica*), and *G. gallus* were 82.03, 75.63, and 75.58%, respectively. More distant similarities were observed with *M. musculus* (76.78%), *H. sapiens* (76.00%), *C. picta bellii* (70.11%), *G. seraphini* (74.77%), *C. harengus* (73.61%), and *O. niloticus* (84.38%) (Figure 4A). The ML phylogenetic tree indicated that *Sox30* from *P. sinensis* showed the highest similarity to other turtle species, followed by mammals, birds, amphibians, and fishes, suggesting that *P. sinensis* shares closer evolutionary relationships with reptiles and occupies a distinct phylogenetic position within the group (Figure 4B).

### 3.3. Expression of Sox3 and Sox30 Genes in Embryonic Gonads and Adult Tissues of P. sinensis

**Figure 5:**
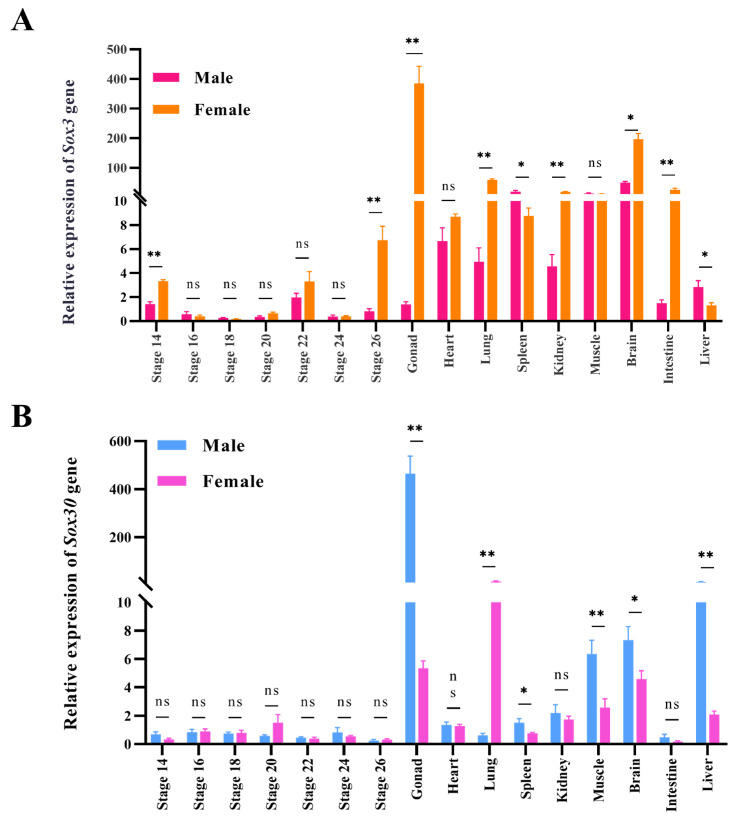
(**A**) Expression of *Sox3* gene in gonads of male and female embryos and adult tissues of *P. sinensis.* (**B**) Expression of *Sox30* gene in gonads of male and female embryos and adult tissues of *P. sinensis*. Notation: ** (*p* < 0.01), * (*p* < 0.05), ns indicates no significant difference. All data are presented as mean ± standard error (n = 3).

RT-qPCR was performed to examine the mRNA expression levels of *Sox3* and *Sox30* in the gonads at different embryonic stages and in various adult tissues of *P. sinensis*. The results showed that *Sox3* in *P. sinensis* exhibited a female-biased expression pattern, with significantly higher expression in the female gonads, kidney, brain, liver, lung, spleen, and muscle compared with those in males (*p* < 0.05). In embryonic gonads of both sexes, the expression level of *Sox3* was relatively low across all stages, and significant sex-dependent differences were only observed at stages 14 and 22 (*p* < 0.01) (Figure 5A). In contrast, *Sox30* displayed a male-biased expression pattern, being expressed at significantly higher levels in male gonads, spleen, muscle, brain, and liver compared with females (*p* < 0.05). However, in embryonic gonads of *P. sinensis*, the expression of *Sox30* remained relatively low across all stages, with no significant differences detected between the sexes (Figure 5B).

### 3.4. Effects of E_2_ and MT Treatments on the Expression of Sox3 and Sox30 Genes in Gonads of P. sinensis Embryonic Gonads at Different Developmental Stages

**Figure 6:**
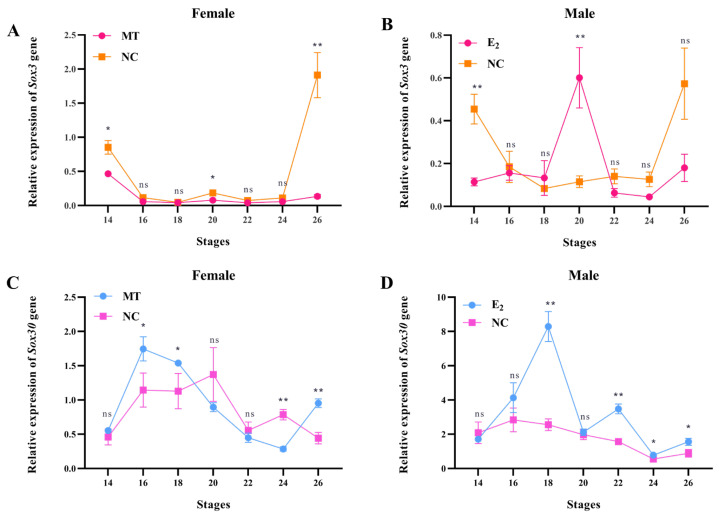
(**A**) Expression of *Sox3* in female *P. sinensis* embryos after MT treatment. (**B**) Expression of *Sox3* in male *P. sinensis* embryos after E_2_ treatment. (**C**) Expression of *Sox30* in female *P. sinensis* embryos after MT treatment. (**D**) Expression of *Sox30* in male *P. sinensis* embryos after E_2_ treatment. Notation: ** (*p* < 0.01), * (*p* < 0.05), ns indicates no significant difference. All data are presented as mean ± standard error (n = 3).

Compared to the control group, MT treatment led to an overall reduction in *Sox3* expression in female embryonic gonads of *P. sinensis*, with significant differences observed at stages 14, 20, and 26 (*p* < 0.05) (Figure 6A). In contrast, E_2_ treatment increased *Sox3* expression in male embryonic gonads of *P. sinensis*, peaking at stage 20 (*p* < 0.01) (Figure 6B). Additionally, MT treatment in female embryos of *P. sinensis* significantly upregulated *Sox30* expression compared with the NC group (Figure 6C). Similarly, E_2_ treatment in male embryos of *P. sinensis* significantly elevated *Sox30* expression compared with the NC group (Figure 6D).

## 4. Discussion

Gonadal differentiation and development is a crucial process in the sex determination of *Testudines*, resulting from the interaction between sex-related genes and endogenous hormones; however, the underlying molecular mechanisms remain poorly characterized [33]. In the present study, we cloned the cDNA sequences of *Sox3* and *Sox30* from *P. sinensis*, with lengths of 1062 bp and 2340 bp, respectively. The CDS were 933 bp and 1659 bp in length, encoding 310 and 552 amino acids, respectively. Systematic analyses of the two genes and their encoded proteins revealed that both *Sox3* and *Sox30* proteins are hydrophilic, with instability indices greater than 40, which is consistent with the flexible structural characteristics typical of transcription factors. The secondary structure prediction indicated that both *Sox3* and *Sox30* predominantly consist of random coils (79.35 and 87.14%, respectively), with limited α-helices and very low proportions of β-sheets, suggesting that their overall conformation is flexible, which facilitates binding with DNA and other regulatory factors [10,34]. Notably, *Sox30* contained more putative phosphorylation sites than *Sox3*, implying that it may be subject to more complex signaling regulation. Comparative amino acid sequence analysis and maximum likelihood (ML) phylogenetic tree construction further revealed that both genes are most closely related to those of turtles, while being most distantly related to those of fish. In summary, these findings suggest that *P. sinensis Sox3* and *Sox30* share common physicochemical properties and secondary structural features but also exhibit distinct differences, which may be associated with their functional divergence in the reproduction and development of this species.

RT-qPCR indicated that *Sox3* expression in the brain and gonads of adult *P. sinensis* was significantly higher than in tissues such as the heart, liver, spleen, and intestine (*p* < 0.05). Previous studies have shown that *Sox3* is expressed in undifferentiated spermatogonia and in the developing hypothalamic–pituitary axis of mammals [35], and is widely involved in neural development and neural stem cell differentiation across vertebrates [36,37]. Notably, the expression of *Sox3* in the ovary was significantly higher than in the testis (*p* < 0.01), exhibiting clear sexual dimorphism. This is consistent with the expression pattern of *Sox3* in various fish species, including *D. rerio* [7] and Malabar red snapper (*Lutjanus malabaricus*) [38]. During embryonic development of *P. sinensis*, *Sox3* expression was significantly higher in female gonads than in male gonads at stage 14 (early gonadal differentiation stage [32] (*p* < 0.01), suggesting that *Sox3* may play a role in female gonadal development. Studies have also shown that in the giant grouper *E. coioides* [39] and *A. japonica* [3], the *Sox3* gene plays a more significant role in ovarian development and oogenesis than in spermatogenesis. RNA-seq analysis of gonadal tissues from male and female *P. sinensis* embryos also revealed that *Sox3* was upregulated in the ovaries, whereas *Sox8* and *Sox9* were elevated in the testes, suggesting a coordinated role of these genes in gonadal development [40]. Further functional studies using knockout or overexpression approaches will be required to clarify the regulatory mechanisms of *Sox3* in gonadal differentiation and development of *P. sinensis*.

In contrast, the expression pattern of *Sox30* is markedly different from that of *Sox3*. In adult *P. sinensis*, *Sox30* is highly expressed in the testis (*p* < 0.05). The expression pattern is highly consistent with those observed in animals such as *L. malabaricus* [38], *O. niloticus* [9], and *M. musculus* [41]. The relatively high expression level of *Sox30* in the brain may be associated with neural development, which is consistent with findings in *D. rerio*, where in situ hybridization localized *Sox30* expression to the midbrain and hindbrain [42]. During the critical stages of embryonic development in *P. sinensis*, the expression level of *Sox30* is relatively low in both female and male embryos. Previous studies have shown that in carp (*Cyprinus carpio*), *Sox30* is localized in spermatocytes and sperm [10]; in *M. musculus*, it is primarily expressed during spermatogenesis after meiosis [43]. Similarly, Tang et al. [44] showed that *Sox30* is localized in spermatocytes, round spermatids, epithelial cells of the efferent duct, sperm, and mature sperm cells in the testes of *O. niloticus* but was not detected in the ovaries. Collectively, these findings suggest that *Sox30* exhibits male-specific expression in *P. sinensis* and likely functions in testis maturation and spermatogenesis, rather than in the early events of sex determination.

In this experiment, after treatment with E_2_ and MT hormones, the expression of *Sox3* and *Sox30* genes in the male and female *P. sinensis* embryos underwent rapid changes during the critical stages of sex determination and differentiation (stages 14–26). Previous studies have demonstrated that exogenous hormones can interact with sex-related genes to influence sex divergence and gonadal development in lower vertebrates, including reptiles [45,46]. In MT treatment female embryos, *Sox3* expression exhibited a downward trend, which may have resulted from the disruption of the endogenous hormonal balance. Specifically, MT stimulation increases androgen levels, antagonizing endogenous estrogen and thereby exerting negative feedback regulation on *Sox3*. Similarly, Deng et al. [47] reported that *Foxl2* expression was significantly downregulated in MT-treated catfish (*Clarias fuscus*). Previous studies have indicated that stages 15–20 represent the critical window for sex differentiation in *P. sinensis* [13,48]. In contrast, E_2_ treatment significantly upregulated *Sox3* expression in male embryos, a pattern comparable to the E_2_-induced response of *Foxl2* in embryos. This suggests that *Sox3* is estrogen-dependent and positively regulated by estrogen receptors, likely playing a role in gonadal development of *P. sinensis* [31].

After MT hormone treatment in female *P. sinensis* embryos, the expression of *Sox30* gene showed an upward trend, which may be closely associated with the shift in gonadal development from ovary to testis, suggesting its important role in sex reversal and masculinization in *P. sinensis*. Previous studies have shown. that in some protandrous fish species, a significant downregulation of *Cyp19a1* expression was observed, leading to a reduction in estrogen synthesis. The disrupting of the positive feedback loop that maintains feminization removes the inhibition on the male-related gene regulatory network. Allowing gonads to develop in the male direction [49]. Studies have shown that *Sox30* is highly expressed in the testes of various vertebrates and plays a role in spermatogenesis [50]. Similarly, in common *C. carpio*, *Sox30* expression was found to be elevated during spermatogenesis and sperm formation [10]. In E_2_-treated male embryos, *Sox30* expression was also upregulated compared with the NC group. This altered expression may reflect a genetically triggered compensatory mechanism or a resilience response, in which males enhance the expression of testis-associated genes to counteract feminization. Supporting this hypothesis, studies in *O. niloticus* have demonstrated that *Dmrt1* positively regulates *Sox30* expression by binding to putative cis-regulatory elements in its promoter region [51]. Similarly, transcriptomic analysis in the South American teleost (*Astyanax altiparanae*) revealed that male-biased genes such as *Dmrt1*, *Sox9*, and *amh* exhibited comparable regulatory mechanisms [52].

## 5. Conclusions

In this study, the full-length cDNAs of the *Sox3* and *Sox30* genes were successfully cloned from *P. sinensis*. Their sequence lengths were 1062 bp and 2340 bp, encoding 310 and 552 amino acids, respectively. Both genes exhibited a close phylogenetic relationship with those from other reptilian species. The *Sox3* gene showed a female-biased expression pattern, while the *Sox30* gene displayed a male-biased expression pattern. Both genes were expressed at relatively low levels during embryonic gonadal development. Exogenous hormone treatments further revealed that MT suppressed *Sox3* but promoted *Sox30* expression in female embryos, while E_2_ enhanced the expression of both genes in male embryos. Taken together, these findings suggest that *Sox3* may be involved in ovarian development, while *Sox30* plays a role in testis development and maturation, potentially contributing spermatogenesis. Both genes are subject to regulation by exogenous hormones. Overall, this study provides novel insights and a theoretical foundation for future research on gonadal differentiation, development, and sex regulation in turtles and other related reptilian species.

## Figures and Tables

**Table 1:** Primers used for cDNA cloning and expression analysis of *P. sinensis*.

Name	Sequences (5′–3′)	Purpose
*PB1* *-F*	GGATCTCATTTGTGAGCCTACATGT	Sex identification
*PB1-* *R*	CCCACAGCTTGCTTTCCWTGTTTAG	
*Sox3*-F	TTGGCCGTGGTGCAGTAGC	cDNA cloning
*Sox3*-R	ATGAGTGTAGAGGTGGAATGGAAA	
*Sox30*-F	GAGGAGAGCCAGAAAGAGGAGC	cDNA cloning
*Sox30*-R	TATTAGTGGGAGTGGGGGTGACAGAA	
*Sox3*-qF	GCAGTACAGCCCCATGATGT	Real-time fluorescence quantification
*Sox3*-qR	GATCATATCCCGCAGGTCC	
*Sox30*-qF	CGAATGCCTGGGCTTTTA	Real-time fluorescence quantification
*Sox30*-qR	GATGGGGTTGCCGTGAAA	
*Ef1α*F	ACTCGTCCAACTGACAAGCCTC	Reference genes
*Ef1α*R	CACGGCGAACATCTTTCACAG	

**Table 2:** Test Comparison Species Sequence Numbers.

Gene	Species	Accession
*Sox3*	*Chelonia mydas*	XP_037764741.1
*Sox3*	*Chrysemys picta bellii*	XP_005294674.2
*Sox3*	*Trachemys scripta elegans*	XP_034636385.1
*Sox3*	*Geotrypetes seraphini*	XP_033802893.1
*Sox3*	*Xenopus tropicalis*	NP_001007502.1
*Sox3*	*Alligator mississippiensis*	XP_006031121.1
*Sox3*	*M. musculus*	NP_033263.2
*Sox3*	*H. sapiens*	NP_005625.2
*Sox3*	*Clupea harengus*	XP_012695760.1
*Sox3*	*D. rerio*	NP_001001811.2
*Sox3*	*Gallus gallus*	NP_989526.1
*Sox3*	*Cyanistes caeruleus*	XP_023782651.1
*Sox30*	*C. picta bellii*	XP_005300115.3
*Sox30*	*T. scripta elegans*	XP_034635714.1
*Sox30*	*Mauremys reevesii*	XP_039340705.1
*Sox30*	*Dermochelys coriacea*	XP_038267805.1
*Sox30*	*G. seraphini*	XP_033783385.1
*Sox30*	*X. tropicalis*	XP_031753986.1
*Sox30*	*A. mississippiensis*	XP_019331538.2
*Sox30*	*O. niloticus*	XP_003447014.1
*Sox30*	*Clupea harengus*	XP_042564328.1
*Sox30*	*M. musculus*	NP_775560.1
*Sox30*	*G. gallus*	XP_414564.1
*Sox30*	*Coturnix japonica*	XP_015731686.1

## Data Availability

The data presented in this study are available on request from the author. Email: xkl954334197@163.com.

## Supplementary Materials

The following supporting information can be downloaded at: https://www.mdpi.com/article/10.3390/genes16111249/s1, Figure S1: Alignment of the cloned *Sox3* sequence from *P. sinensis* with the NCBI reference sequence (XM_006115453); Figure S2: Alignment of the cloned *Sox30* sequence from *P. sinensis* with the NCBI reference sequence (XM_006138296).
